# A review on edible vaccines and their prospects

**DOI:** 10.1590/1414-431X20198749

**Published:** 2020-01-24

**Authors:** B. Gunasekaran, K.M. Gothandam

**Affiliations:** School of Bio Sciences and Technology, Vellore Institute of Technology, Vellore, Tamil Nadu, India

**Keywords:** Vaccines, Plants, Algae, Probiotics, Edible vaccine

## Abstract

For a long time, vaccines have been the main mode of defense and protection against several bacterial, viral, and parasitic diseases. However, the process of production and purification makes them expensive and unaffordable to many developing nations. An edible vaccine is when the antigen is expressed in the edible part of the plant. This reduces the cost of production of the vaccine because of ease of culturing. In this article, various types of edible vaccines that include algal and probiotics in addition to plants are discussed. Various diseases against which research has been carried out are also reviewed. This article focused on the conception of edible vaccines highlighting the various ways by which vaccines can be delivered.

## Introduction

More than one million people die each year of infectious diseases. Fifty percent of these diseases are caused by pathogens infecting the mucosal membrane of the mammalian host (1). The challenge today is to find unique and innovative vaccines that can target pathogens and infections at various stages.

Vaccines are biological preparations that improve our immunity. The concept of vaccination was first put forth by Edward Jenner in 1796 for small pox. Vaccination is the process by which the body is made ready to face and fight off new infections. This way of treatment is in direct contrast to the classical way of treatment, which usually is done after the onset of a specific disease. Vaccines not only prepare us against any future infection but also immunizes us against those infections for a very long time. The major drawback until now has been the production process. Vaccines are generally produced by industrial processes, thus making them expensive and inaccessible in developing countries (2,3). For this very reason, edible vaccines are seen as ideal replacements for conventional vaccines. Edible vaccines are generally antigen-expressing plants, thus requiring basic knowledge on agriculture and how to grow plants to be produced. Also, in edible vaccines, the process of purification and downstream processing, which make conventional vaccines costly, are eliminated (4–6).

Post translational modifications that generally occur in eukaryotic expression systems may positively affect the immunogenicity of the expressed antigen (7). However, as per the experiment carried out by Giersing et al. (8), post-translational modifications do not always enhance the effectivity of the vaccine. Expression of a protein in a prokaryotic system like *E. coli* also showed equal immunogenicity. For a long time, mammalian recombinant expression systems were used to express such proteins even though mammalian systems are very difficult to handle and expensive. They also have low expression levels making them a bad choice to be used as a protein expression platform (9).

This article focuses on the evolution of edible vaccines over the years and the various prospects it holds as technology keeps evolving.

- The evolution of vaccines has led to the discovery of new forms of vaccination that are effective and cover a wider array of disease.
- Live-attenuated vaccines: these are considered the original and 1st vaccines. Here, the weakened form of a live infectious organism is used as a vaccine.
- Inactivated vaccines: these are vaccines where the debris of the dead organism is used as a vaccine.
- Toxoid vaccines: the toxin generated by the organism is used as the vaccine. Toxoid vaccines focus on preventing the ill effects from the infection rather than the infection itself.
- Biosynthetic vaccines: as the name suggests, these vaccines are man-made and have very similar shape and properties to the infectious organism.
- DNA vaccines: plasmid DNA with sequences encoding the antigen. This plasmid DNA is then introduced directly to a specific muscle or tissue where it is expressed.
- Recombinant vaccines: vaccines where a recombinant plasmid with the gene encoding the antigen is expressed in bacteria. This protein is then purified and used as vaccine.
- Edible vaccines: the edible part of a plant is genetically modified to express antigens, thus eliciting an immune response upon consumption.

## Concept of edible vaccines

Edible vaccines are created by introducing the desired gene into a plant to manufacture the encoded protein. The coat protein of a specific virus or bacteria that has no pathogenicity is used for transformation. Table 1 shows the various transformation techniques used for plant, algal, and bacterial vaccine carriers. Edible vaccines can be very easily scaled up. For example, the entire population of China could be vaccinated by producing edible vaccines in just 40 hectares of land. Chance of contamination by plant pathogens is very low or rather insignificant as plant pathogens are not capable infecting human beings (10). Edible vaccines against various diseases such as measles, cholera, foot and mouth diseases, and hepatitis B, C, and E are produced in plants like banana, tobacco, potato, etc. (11).

**Table 1:** Transformation techniques in plants, microalgae, and bacteria.

Transformation method	Plant	Microalgae	Bacteria	Reference
Agrobacterium mediated gene transfer	•	•		(14–16)
Biolistic method/ Gene gun	•	•		(17–19)
Electroporation	•	•	•	(20–23)
Glass beads		•		(24,25)
Electrospray			•	(26)
Heat-shock method			•	(90)

## Mechanism of action of edible vaccines

**Figure 1:**
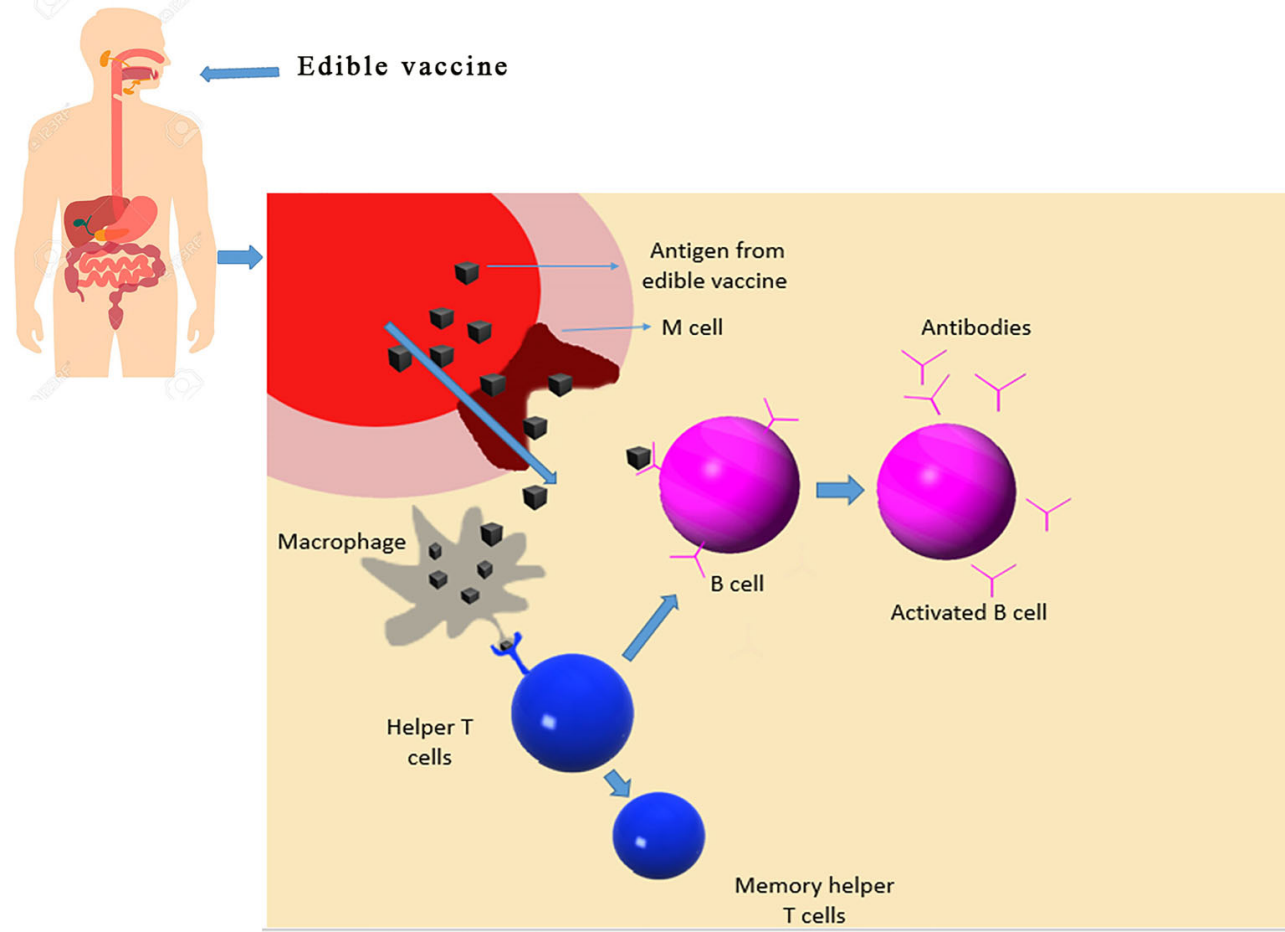
Mechanism of action of edible vaccines.

Edible vaccines are required to induce the activation of the mucosal immune response system (MIS). The MIS is the first line of defense as it is where human pathogens initiate their infection. Mucosal surfaces are found lining the digestive tract, respiratory tract, and urino-reproductive tract. There are multiple ways by which the antigen can enter the gut mucosal layer, namely by M cells and macrophages. Macrophages are usually activated by interferon gamma. This activation leads to the macrophages presenting fragmented peptides to the helper T cells that further produce antibodies (12). M cells are another way by which the antigens are transported to the T cells. The antigenic epitopes are then present on the APC surface with the assistance of helper T cells, which then activate B cells. Activated B cells then migrate to the mesenteric lymph nodes where they mature into plasma cells, which then migrate to mucosal membranes to secrete immunoglobulin A (IgA). IgA then forms the secretory IgA, which is then transported into the lumen. Production of secretory IgA is another complex event since 50% of secretory IgA (sIgA) in gut lumen is produced by B1 cells in the lamina propria in a T-cell-independent fashion. These sIgA are polyreactive and usually recognize the foreign antigens. In the lumen, the sIgA neutralizes the invading pathogen by reacting with the specific antigenic epitopes (as shown in Figure 1) (13). The most common problem most oral vaccines/therapeutics face is the tolerance towards the vaccine in the gut. This problem can be overcome by some methods:

- Immune suppression by using triamcinolone. However, this has to be done in small amounts so as prevent any major health concerns or even fatality.
- Increasing the dosage of the vaccine significantly can often lead to jump starting the immune response.
- Multiple doses over a specific period of time as suggested by Silin and Lyubomska (14).

## Edible plant vaccines

Plants started gaining focus as recombinant expression systems in the late 1980's. Plants have a very important advantage over mammalian expression system: they require no external carbon source as they are fueled by photosynthesis (15,16). Another major advantage a plant system has on a mammalian system is the absence of contamination by mammalian pathogens. These advantages specifically make the production of antigens, vaccines, and other eukaryotic proteins in plants more interesting (17,18).

### What makes a candidate plant?

Candidate plants are those plants that are most suitable for edible vaccine production. There are a number of factors that make a plant a good edible vaccine candidate.

- Must have long shelf life. The plant or the edible part of the plant has to be stored for a long time without degradation. Cereals such as rice, maize, and wheat are great examples of such plants.
- Must grow quickly. Fruits or vegetables that usually are produced on trees are considered bad candidates as they take a long time to grow and mature, whereas plants such as tobacco and tomato have fast growth time.
- Easy transformation. Plants on which considerable research has been carried out and transformation techniques optimized are very good candidate plants.

Plants, unlike other expression systems can be scaled up to need, making it easily available to the masses.

### Plants commonly used as candidates

Plants with the above-mentioned qualities are generally selected to be edible vaccines. Plants such as tomato, tobacco, rice, and maize are widely used for this purpose.

#### Tobacco

Tobacco was a previously used model plant. It has many advantages such as fast growth, large number of seeds per generation and it is perennial. Tobacco has been used as an edible vaccine candidate extensively (19).

#### Potato

Potatoes are tubers that are widely eaten all around the world and very affordable. A large amount of data on generic manipulation is available, thus making optimized protocols available. The one major disadvantage of using potato is that it requires cooking before consumption. Cooking can denaturate the antigen (20).

#### Rice/maize

Rice and maize are cereals that are staples in many countries. The main reason why rice and maize are attractive as candidate edible vaccines is because they can be stored without refrigeration for a very long period of time. But the disadvantage with cereals is that they take relatively long periods of time and require perfect conditions to grow (21).

#### Tomato

Tomato is another plant that is widely used and is a popular choice for use as an edible vaccine. It grows relatively quickly and tastes good, thus having a broader range of consumers. The major disadvantage with tomato is that it spoils rapidly after ripening (21).

To date, various plants have been used to express foreign antigens in their edible parts and then used as edible vaccines. Table 2 shows the research carried out, some of which are explained in detail below.

**Table 2:** Edible plant vaccines for various diseases in human clinical trials.

Disease	Host plant	Reference
Hepatitis B	Lettuce	(37)
	Potato	(38)
Cholera	Rice	(39,40)
Influenza	*Nicotina benthamiana*	(41)
	*Nicotina benthamiana*	(42)
	*Nicotina benthamiana*	(43,44)
Rabies	Spinach	(45)
ETEC	Potato	(46)
	Maize	(47)

ETEC: enterotoxigenic *Escherichia coli* infection.

#### Norwalk disease

Norwalk disease is caused by Norwalk virus, a member of the caliciviridae family (22). It causes acute gastroenteritis in human beings. Norwalk virus genome was cloned and that has facilitated the production of various vaccines (23). Norwalk virus capsid protein was expressed in insect cells. The resulting protein lacked the viral RNA thus making it non-pathogenic (24). The particles closely resembled an authentic Norwalk virus both antigenically and morphologically (25). Plant expression vectors pNV101, pNV102, and pNV140 were constructed by Mason (5). These plasmids were then transformed by using *Agrobacterium tumifaciens* LBA4404 by the freeze-thaw method. The Norwalk virus coat protein (NVCP) was then quantified with ELISA (23) using rabbit anti (i-rNV) serum diluted 1:10000 in 0.01 M PBS. The recombinant Norwalk virus-like particles were extracted from plant tissue and then purified (5). This purified protein was then quantified and qualified using anion exchange chromatography, SDS PAGE, and western blotting. Mice were fed with the recombinant proteins and they showed production of humoral and mucosal antibody responses.

#### Hepatitis B

Hepatitis B is an infectious disease caused by the hepatitis B virus (HBV), which affects the liver. It rarely leads to death. The hepatitis B surface antigen (HBsAg) was expressed in transgenic lupin callus by feeding the mice with transgenic lupin callus tissue and HBsAg specific antibodies. The DNA that encodes for the surface antigen of HBV was cloned. The plasmid pROK25 carrying the HBsAg coding sequence was electroporated into *Agrobacterium tumifaciens* LBA4404 (26) and C58. C58 was used for transforming seedlings of yellow lupin and LBA4404 was used on the lettuce plant. Both transformations were successful and the protein was extracted and analyzed by plotting a standard curve based on different concentrations of HBsAg (27). The transgenic lupin tissue was fed to mice and human volunteers were fed with transgenic lettuce. ELISA was carried out on both the mice and the human volunteers' serum samples (28). Both samples showed antibody titers.

#### Cholera

Cholera is a bacterial disease caused by *Vibrio cholerae,* a Gram-negative, comma-shaped bacteria that causes acute watery diarrhea by colonizing the small intestine and producing an enterotoxin, cholera toxin B (CTB). CTB acts as a potent mucosal immunogen when taken orally (29). This is the result of the CTB binding to the eukaryotic cell surfaces via the GM1 ganglioside receptors present on the epithelial surface of the intestines, thus eliciting a mucosal response to pathogens (30). Immune response is enhanced when it is chemically coupled to other antigens (31).

In an experiment carried out by Daniell et al. (32), the construction of the chloroplast expression vector, pLD-LH-CTB, was carried out. The CTB production in *E. coli* was analyzed using immunblot assay. Then, the plasmid DNA (pLD-LH-CTB) were bombarded into the *Nicotina tobacum* leaves. The transformed leaves were cut and grown in a medium containing a selection marker, in this case streptomycin (33). PCR analysis was done followed by southern blot analysis. Western blot analysis and ELISA were used to quantify the amount of CTB protein produced. Finally, GM1 ganglioside assay was done showing that both the chlorophyll-synthesized CTB and the bacterial CTB demonstrated a strong affinity for GM1 ganglioside (33). High levels of constitutive expression of CTB in transgenic tobacco do not affect the growth rate, flowering, and seeding, unlike when expressed in nuclear genome (34).

## Edible algal vaccines

Algal edible vaccines are similar to plant edible vaccines. Algae are sometimes referred to as single-celled water-borne plants. There are very few strains of algae that are considered edible for human beings and are capable of being genetically engineered to deliver antigens against various diseases. The usage of algae has many advantages such as:

- Microalgae are much easier to be genetically modified, thus showing higher expression levels of foreign genes (35).
- Algal vaccines are relatively cheaper compared to those produced by plants.
- Algae are a potential source of food for many species including human beings (36).
- Microalgae are resistant to animal pathogens, thus making them a very good mode of vaccine production.

Table 3 shows all the diseases against which microalgae have been used as a method of vaccination by direct consumption. A few important examples are discussed in detail below.

**Table 3:** Edible algal vaccines for various diseases.

Disease	Host algae	Reference
Malaria	*Chlamdomonas reinhardtii*	(64–67)
Hepatitis B	*Dunaliella salina*	(68)
	*Phaeodactylum tricornutum*	(69)
Foot and mouth disease	*Chlamydomonas reinhardtii*	(70)
Classical swine flu	*Chlamydomonas reinhardtii*	(71)
White spot syndrome	*Chlamydomonas reinhardtii*	(72)
Staphylococcus aureus	*Chlamydomonas reinhardtii*	(73)
Human papilloma virus	*Chlamydomonas reinhardtii*	(74)
Hypertension (angiotensin II)	*Chlamydomonas reinhardtii*	(75)

###  

#### Foot and mouth disease

Foot and mouth disease virus (FMPV) is a major disease that infects livestock (37) and it has been under control mainly due to vaccinations. Both inactivated and attenuated vaccines are used but are generally not considered to be completely safe. The structural protein of the FMDV, VP1, has critical epitopes that have the ability to produce antibodies (38). Cholera toxin B subunits were used as they are very effective at acting as mucosal adjuvant that can bind to the intestinal epithelial surfaces through the GM1 gangliosides receptors. The plasmid pACTBVP1 was transformed by means of biolistic bombardment into the microalgae *Chlamydomonas reinhardtii*. After transformation, it was incubated under dim light until the cells turned yellow as reported by Suzuki et al. (39). The selected transformants (streptomycin resistance) were analyzed by PCR with ChIL primers. The PCR products were then analyzed by southern blotting. The presence of the CTBVP1 fusion protein was analyzed by western blotting. ELISA was carried out for quantitative analysis. The fusion protein showed weak but significant binding affinity for GM1 ganglioside. The research by Sun et al. (40) showed that *Chlamydomonas* expressed CTBVP1 in large quantities. It also showed that this fusion protein bound to the GM1 ganglioside, meaning it could be used as a potential mucosal vaccine source.

#### Hepatitis B

Hepatitis B is one of the most widespread chronic diseases that infects up to 350 million people worldwide (41). Hepatitis B surface antigen HBsAg has been used as a vaccine for quite some time. HBsAg is usually isolated from high-titer patients. Currently, the hepatitis vaccine is being produced mainly in yeast (42,43). The HBsAg antibody was expressed in an algal expression vector, *Phaeodactylum tricornutum*. The results of the study showed that the human antibody CL4mAb was expressed and assembled in the endoplasmic reticulum of the microalgae. When the same antibody was expressed in the plant *Nicotina tobacum*, it showed much lower expression levels (44). Protein degradation, which was reported to be a major problem in plants (45,46), was not found when the same protein was expressed in *P. tricornutum*. ELISA assay with whole protein extract and also with purified protein from the algae showed that this antibody binds to the antigen HBsAg very effectively. In addition to producing these antibodies, the HBsAg antigen was expressed in *P. tricornutum* (47). HBsAg is very commonly used as a vaccine against hepatitis B. When expressed in the microalgae, 0.7% of the total soluble proteins was HBsAg. This antigen was recognized by the algae-produced antibody and by the commercially produced antibody.

In another study, Geng et al. (48) showed the transformation of the *HBsAg* gene into the algae *Dunaliella salina.* This was carried out by electroporation (49). Chloramphenicol-resistant strains were selected and checked by molecular analysis. Successful integration of the *HBsAg* gene into *Dunaliella salina* genome was verified by PCR and southern blotting. By carrying out ELISA, it was found that a large quantity of HBsAg protein was expressed by *D. salina.* This HBsAg was found to have immune activity.

#### Classical swine flu

Classical swine flu virus (CSFV) is a highly contagious virus that leads to classical swine fever (50,51). Even though vaccines are the leading prevention method against CSFV, attenuated vaccines and C-strain vaccines have been reported to have lost their ability to differentiate between infected and a vaccinated animal (52). The E2 protein has major antigenic properties and neutralizes its respective antibodies. In research carried out by He et al. (53), this E2 protein from the CSFV was expressed in *Chlamydomonas reinhardtii.* Immune experiments were carried out on an animal model in order to check the immunogenicity of the expressed protein. There was an increase in serum antibody against CSFV when the extract was administered subcutaneously.

#### Staphylococcus aureus infection

*S. aureus* is a Gram-positive bacterium. It belongs to the group of bacteria called firmicutes. *S. aureus* is a human pathogen that infects the nasal mucosa and the skin (54). It is responsible for bacteremia, which is the cause for secondary infections such as endocarditis, pneumonia, meningitis, etc. (55). Dreesen et al. (56) reported that the fibronectin-binding protein expressed by *S. aureus* is very important for its pathogenicity, and it is fused with the cholera toxin B. The fibronectin binding protein adheres to the extracellular matrix of the host (57). The CTB improved the antigen-specific immune response (58). The CTB-D2 fusion antigen was codon optimized and expressed in the chloroplast of the microalgae *C. reinhardtii*. The CTB-D2 antigen was resistant to conditions mimicking the stomach environment and at low pH. It also bound to the GM1 ganglioside and triggered a systemic and mucosal immune response. CTB-D2 antigen-expressing algae were lyophilized and then fed to mice, which were protected against lethal doses of *Staphylococcus aureus.*

#### Malaria

Malaria is a disease that is caused by the parasitic protozoa *Plasmodium falciparum.* It is transmitted by a mosquito bite. Annually, close to 100 million deaths occur, with at least 300 to 500 million infections (59,60). The most advanced and recent vaccine being used against malaria is specifically against the sporozoite. This vaccine is designated RTS, S/ASO2A. In a study carried out by Dauvillée et al. (61), high levels of granule bound starch synthase (GBSS) bound to starch, which fuse to three malarial vaccine candidates, and were then expressed in the microalgae *C. reinhardtii.* It was shown that the amount of starch-antigen that was accumulated in the chloroplast of the algae was sufficient to provide protection against otherwise lethal doses of *Plasmodium falciparum* in mice. This inhibition was observed because of the blockade of erythrocyte invasion. In this study, *C. reinhardtii* was used as the starch in its chloroplast, which stabilized the vaccine over longer periods of time. Also, this alga has a GRAS (generally regarded as safe) status and is much easier to scale up and cultivate.

In a study by Gregory et al. (62), the malarial subunit vaccines pfs25 and pfs28 were expressed in *C. reinhardtii.* Both these subunits are structurally complex malaria transmission-blocking vaccine candidates. The algae-produced pfs25 and pfs28 were found to have structural similarity to the native pfs25 and pfs28. This makes the algal expression system the only system to express these 2 proteins in an unmodified glycosylated form. The similarity in structure was identified using monoclonal antibodies that only bind to conformationally correct pfs25 and pfs28. In yeast homologues of pfs25, the disulphide bonds were found to be lacking (63), but the algal expression system expressed the pfs25 with disulphide bonds. It was shown that a-pfs25 but not a-pfs28 showed significant transmission-blocking capabilities, which is consistent with previous works (64,65).

#### Human papilloma virus

The human papilloma virus is responsible for almost 6.1% of all cancer cases worldwide. Of those, 99.7% are agents responsible for cervical cancer (66). More than half the cases are caused by HPV16 (67). Conventional therapies are not effective against cervical cancer tumors, are often toxic, and can lead to recurrences (10-20% possibility). The hr- HPV-E7 oncoprotein, which is involved in the malignant cellular transformation, is the perfect candidate for development of therapeutic vaccines (68). In the work done by Demurtas et al. (69), the HPV-E7 protein in its attenuated form was expressed in the microalgae *C. reinhardtii.* It showed positive results in preclinical animal models. This antigen has been thus far analyzed for biochemical and physical studies, but the expression in algae has now opened new possibilities (70,71). Future works could see the overexpression of this antigen in algae, for direct use as vaccine against HPV.

## Probiotics as edible vaccines

Genetically modified bacteria have been used as vaccines in three different ways. First, live vaccines are mutated or have a gene deleted, thus hindering their ability to infect mammalian cells (72). Second, by producing proteins and using bacteria as a low-cost protein factory. These proteins can then be purified and used as vaccines (73). Third, by ingestion of a bacterium expressing a foreign antigen. Usually commensal bacteria are chosen for this purpose as they pose no threat to the human system (74). The bacterial species that are most commonly used for vaccine delivery are *Listeria monocytogenes, Salmonella spp, Yersinia enterocolitica,* and other commensal organisms.

### Examples of bacterial carriers

Most of these organisms are either human pathogens or commensal microorganisms. Table 4 shows some of the research that has been carried out on this subject. Important examples are discussed in detail below.

**Table 4:** Live bacterial edible vaccines.

Carrier organism	Disease	Reference
*Listeria monoctogenes*	Influenza	(75)
	HIV	(76)
*Streptococcus gordonii*	HIV	(89)
*Lactobacillus casei*	Anthrax	(87)

#### Listeria monocytogenes

*Listeria monocytogenes* is a Gram-positive bacterium that mediates cell response against its own proteins. What makes *L. monocytogenes* special is its ability to breach into the cytoplasm of the host, thus allowing the recombinant protein into the antigen-processing pathway. This makes it very effective in clearing bacterial, viral, and parasitic pathogens, and tumors (75,76). Mutations in the virulence-related genes are exploited to make suitable vaccine carriers. They are also known to protect against tumors by producing tumor-associated antigens.

#### Salmonella spp

*Salmonella* is a rod-shaped Gram-negative bacterium that is an intracellular pathogen and is restricted to the endosomal compartment of eukaryotic cells. It resists non-specific killing mechanism of the host cell (77). Non-reverting mutations that critically affect the virulence of *Salmonella* are introduced. This makes them very good vaccine carriers (78,79). These mutants are exceptional vaccine carriers for other pathogenic antigens such as viral, bacterial, parasitic, and tumors (80,81), and they stimulate strong local and systemic immune responses.

#### Yersinia entercolitica

*Y. entercolitica* is a rod-shaped Gram-negative bacterium. It usually infects animals but can also infect human beings. It usually infects the host intestinal tissue resisting the clearance mechanism of the host. The presence of a virulence plasmid is what makes *Y. entercolitica* invasive (82). This plasmid encodes for the synthesis of several of the virulence determinants. Recombinant strains of *Y. entercolitica* that express a foreign antibody show a strong mucosal and systemic immune responses (83,84). Antibody production is triggered even in the respiratory tract apart from the intestines.

#### Commensal microorganisms

Microorganisms that are present on the surface, covered by epithelial cells like the gastrointestinal tract, respiratory tract, skin, vagina, etc are generally termed commensal microorganisms. These organisms are considered beneficial to the host and the host provides an ecosystem for the microorganism to flourish. Commensal strains include *Streptococcus gordonii, Lactobacillus spp, Staphylococcus spp,* etc. In research by Fischetti et al. (85), it was observed that Gram-positive commensal strains tended to anchor themselves. This anchorage led to the recombination of the anchoring sites of the commensal bacteria with the foreign antigen. However, it was also observed that not all strains of commensal bacterial showed a similar trend.

#### Lactobacillus

is one of the most common commensal strains present in the gut and genitourinary tract. They also make very good mucosal vaccine candidates because of their large variety of immunomodulatory and biological properties (86). Various *Lactobacillus-*based vaccines have been tested and are shown to have immune responses against the antigen (87). Various antigens of human pathogens have been expressed in *Streptococcus gordanii* also (88,89 [Bibr B90]).

Many human pathogens are known to enter the human system through the genital mucosa. For elicitation of an immune response at a specific location, *Lactibacillus* and *S. gordanii* can be used as an effective method of vaccination against sexually transmitted diseases (89).

## Conclusion

Edible vaccines are much safer and cheaper alternatives to traditional vaccines. As any edible plant/algae, they can make scaling up so much easier. The problem with edible vaccines is the notion that genetically modified crops are bad, which prevails in many developing nations. With the ever growing and evolving technologies, genetically modified crops are getting safer than ever. There have been reports of laboratory-synthesized meat that can act as replacements for normal meat. In the near future, such meat can also be modified to deliver vaccines of interest upon consumption. With edible vaccines popularized properly and distributed around the world, many diseases can be eradicated and millions of lives can be saved.
